# Microglia: A Potential Drug Target for Traumatic Axonal Injury

**DOI:** 10.1155/2021/5554824

**Published:** 2021-05-20

**Authors:** Xin Huang, Wendong You, Yuanrun Zhu, Kangli Xu, Xiaofeng Yang, Liang Wen

**Affiliations:** ^1^Department of Neurosurgery, First Affiliated Hospital, Zhejiang University School of Medicine, China; ^2^Emergency and Trauma Center, First Affiliated Hospital, Zhejiang University School of Medicine, China

## Abstract

Traumatic axonal injury (TAI) is a major cause of death and disability among patients with severe traumatic brain injury (TBI); however, no effective therapies have been developed to treat this disorder. Neuroinflammation accompanying microglial activation after TBI is likely to be an important factor in TAI. In this review, we summarize the current research in this field, and recent studies suggest that microglial activation plays an important role in TAI development. We discuss several drugs and therapies that may aid TAI recovery by modulating the microglial phenotype following TBI. Based on the findings of recent studies, we conclude that the promotion of active microglia to the M2 phenotype is a potential drug target for the treatment of TAI.

## 1. Introduction

Traumatic brain injury (TBI) is a leading cause of disability and mortality among young patients worldwide [1, 2]. Neuroinflammation accompanying the activation of microglia and other effector cells following TBI has been suggested as an important mechanism of secondary TBI [3–7]. Microglia are major immune response cells in the central nervous system (CNS). In addition to robust post-TBI inflammatory responses, neuroinflammation can also be long-lasting, leading to persistent neural injuries that impede repair, or even enhance neurodegenerative disease risk [8–13]. Such inflammatory responses can also include the recruitment of immune cells from the blood, mainly infiltrating macrophages [14]. Active microglia can transform to the M1 phenotype, to secrete proinflammatory and cytotoxic mediators that mediate neuronal dysfunction and cell death, or to the M2 phenotype, to participate in phagocytosis and secrete anti-inflammatory cytokines and neurotrophic factors that are important for neural protection and repair [3, 15, 16].

Most studies on the effects of microglia following TBI have examined focal injury, including cerebral contusions and lacerations. However, axons in white matter are also highly susceptible to injury, making traumatic axonal injury (TAI) one of the most common pathologies of TBI and a critical factor for prognosis [17–21]. Diffuse axonal injury (DAI), which occurs mainly in the corpus callosum, thalamus, and brain stem, has been documented to be a leading cause of mortality and severe conscious disturbance following severe TBI [18, 19, 22]. Therefore, the objective of this review was to elucidate the relationship between neuroinflammation and TAI, and discuss microglia as therapeutic targets for TAI.

## 2. Microglia and TAI

Microglial reactions after TBI have been well established, and microglial assembly in association with TAI has also been documented [23–26]. The most common sites of microglial clustering following TBI are the corpus callosum, internal capsule, and brain stem, where axonal injuries tend to be concentrated [23, 27]. To determine the relationship between axonal injury and activated microglia/macrophages (CD68+ cells), Wilson et al. [27] analyzed paraffin-embedded materials from the pons of head injury patients with DAI-linked disability, and found that CD68+ cells were colocated with terminal deoxynucleotidyl transferase dUTP nick-end labeling- (TUNEL-) positive staining in the corticospinal tracts and the medial lemnisci, which showed Wallerian degeneration. This spatial relationship between axonal injury and CD68+ cells suggests that microglia/macrophages are likely to be involved in TAI. Unlike focal cerebral injury, axonal injury occurs mainly in the corpus callosum, internal capsule, and brain stem, where the blood-brain barrier (BBB) is relatively intact, and the number of infiltrating macrophages is very limited. In further studies that analyzed brain samples from TBI survivors, patients who had survived 12–13 years post-TBI showed immunoreactivity that was attributed to brain tissue microglial/macrophage activation (CD68+ and ionized calcium-binding adapter molecule- (Iba-) 1+) [28, 29]. And the activated microglial/macrophage was associated with the *β*-amyloid precursor protein (APP) immunoreactivity, which represented axonal transport impairment and was a biomarker for axonal injury [28]. These findings indicate that activated microglia/macrophages can persist for long periods post-TBI at axonal injury sites.

Recent studies have shown that microglia in white matter are activated rapidly after TBI, in a manner associated with axonal injury. In a confocal 3D analysis of the physical relationship between microglia and DAI in diffuse mild TBI in minipigs, a significant increase in the number of activated microglia (Iba-1+) was observed at 6 h after TBI, accompanied by APP+ proximal axonal swelling, especially in the thalamus [30]. However, microglial activation in white matter may be delayed compared to that in grey matter. Csuka et al. [31] reported that activated microglia become apparent in white matter regions including the corpus callosum, internal capsule, and optic tract from 24 to 48 h and up to 2 weeks after TBI, whereas such microglial changes were detectable via OX6+ in grey matter nuclei as early as 4 h after TBI. In a human study, Oemichem et al. [32] also reported delayed microglial accumulation at the site of axonal injury compared to focal cerebral injury in the cortex. They performed immunohistochemical double-labeling to demonstrate axonal injury using the *β*-APP antibody and microglia using the CD68 antibody simultaneously in 40 individuals who had survived from 3 h to 15 days post-TBI, and found that microglial/macrophage clusters in areas of axonal injury accumulation were sporadic and did not occur until 4 days post-TBI.

Besides, imaging examinations also revealed the close relationship between activated microglia and TAI in the acute phase. In a study of the effects of controlled skull impact at the bregma on TAI induction in mice, magnetic resonance diffusion tensor imaging (DTI), which is used to detect axonal injury in neural axons [33], showed significant axonal damage in the corpus callosum, accompanied by hypertrophic microglia, characterized by CD11b immunohistochemistry, through post tissue analysis after impact [34]. A study of repetitive mild TBI in mice used DTI and diffusion kurtosis imaging (DKI), and obtained similar results, showing that activated microglia, detected as CD11b+, accompanied axonal injury after TBI [35].

After the acute phase of TBI, progressive neuroinflammation is associated with sustained microglial activity. Subacute or chronic neuroinflammation is more apparent in white matter than in grey matter [36, 37], and probably leads to long-term neural function damage. Significantly higher immunoreactive microglial activity for galectin-3/mac-2-ir, a myelin degradation marker, was observed within the injured corpus callosum of mice with midline closed-skull injury than in that of sham mice at 28 days post-TBI [36]. Another study examined the effect of controlled cortical impact (CCI) on TBI induction and observed activated microglia, detected as ED1+, along the entire injured side of the corticospinal tract (CST) at 2 months post-TBI; the CST exhibited obviously degenerating myelinated axons [38], and activated microglia of the hindbrain pyramids intermingled with degenerating CST nerve axons. Several studies using animal models have reported that microglial activation in white matter is correlated with chronic axonal injury post-TBI [39–42]. Human studies have also shown that sustained microglial activity, which is closely related to chronic neuroinflammation, leads to axonal injury [13, 43–46]. Based on positron emission tomography (PET) and magnetic resonance imaging of the PET ligand [^11^C](R)-PK11195(PK) in TBI patients, microglial activity can persist for up to 17 years post-TBI [37]. In the chronic phase after TBI, no increase in PK binding was observed at the original site of focal cerebral injury, but there was a significant increase at the thalami, occipital cortices, and posterior limbs of the internal capsules, where axonal injury was obvious. In a study that examined microglial density and morphology in brain sections of TBI patients 10–47 years post injury, TBI survivors (>3 months) exhibited extensive, densely packed, CR3/43- and/or CD68-immunoreactive microglia in the corpus callosum combined with white matter degeneration, compared to age-matched, uninjured control participants. These reactive microglia were present in 28% of patients with survival of 1–18 years post trauma [44].

Thus, as the main CNS scavengers, microglia are activated rapidly post-TBI, and they are probably closely related to TAI. The effects of microglia post-TBI are complex, and the mechanisms leading to TAI have not yet been elucidated. For example, in addition to neuroinflammation induction, activated microglia clear myelin debris which is a proinflammatory mediator produced by primary TAI [8, 47, 48], and contribute to anti-inflammatory effects; whereas, long-term activated microglia at the TAI site can cause neuroinflammation with other effector cells, which contributes to chronic degenerative changes [44] such as chronic traumatic encephalopathy (CTE), a frequent consequence of repetitive mild TBI [49]. Focal cerebral injuries are seldom associated with mild TBI, but can lead to diffuse axonal injuries marked by *β*-APP [50]. For this reason, TAI is often analyzed based on mild TBI. Repetitive mild TBI is common in specific populations such as boxers or American football athletes. In patients with chronic neuroinflammation, repetitive mild TBI leads to CTE [49, 51], which is typically characterized by pathological changes such as activated microglia/macrophages in white matter, accompanied by axonal injury [10, 49, 51–53]. Because mild TBI is associated with the infiltration of small numbers of macrophages through the broken BBB, these findings indicate that microglia are probably the key to the relationship between TBI and CTE and other neurodegenerative diseases.

**Figure 1:**
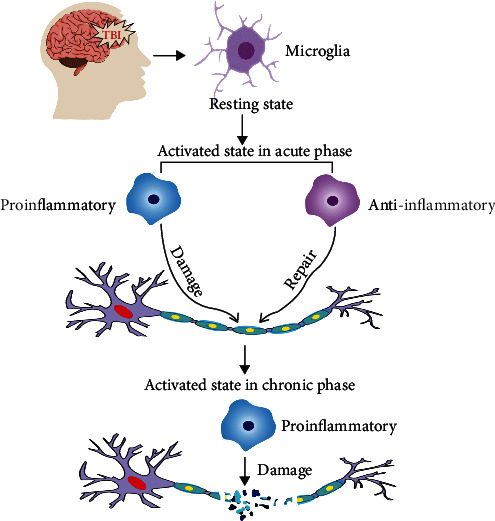
Microglia and TAI: traumatic brain injury will activate microglia rapidly, and microglia will transform from the resting state to the activated state. In the acute phase, activated microglia include proinflammatory and anti-inflammatory phenotypes which will play a role in axonal damage and repair, respectively. While in the chronic phase, activated microglia are mainly proinflammatory and will take part in the long-term process of axonal injury after TBI.

Based on these studies, activated microglia are closely related to acute or chronic TAI and are therefore likely to be an important cause of axonal injury (Figure 1).

## 3. Post-TAI Microglial Polarization

Microglia are important cellular components of the CNS immune system, accounting for 0.5%–16.6% of all CNS cells [54, 55]. In healthy brains, microglia remove debris and metabolic products through phagocytosis without changing phenotype, thereby maintaining the microenvironment. When injuries occur, microglia become reactive, causing significant changes in their morphological features and gene expression levels [3, 14, 56–58].

**Figure 2:**
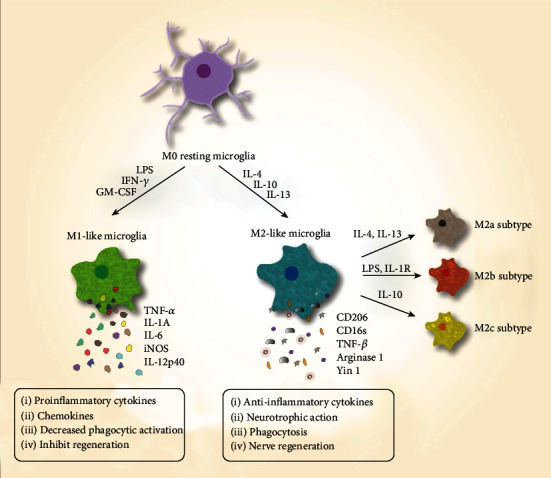
Active microglial phenotypes: microglia can be activated by different types of cytokines. Active microglia have two polarization phenotypes, M1 and M2 [3, 4, 57].

Active microglia have two different polarization phenotypes, M1 and M2 (Figure 2).

M1 activation transforms microglia to potent proinflammatory effector cells, which secrete proinflammatory cytokines including IL-1*β*, IL-6, TNF-*α*, IFN-*β*, and COX-2. Microglial M1 activation occurs through three main pathways: LPS, IFN-*γ*, and GM-CSF [56, 59, 60].

In contrast, M2 activation transforms microglia to anti-inflammatory effector cells that inhibit inflammatory reactions and promote tissue repair. M2 polarization can be divided into at least three subtypes: M2a, M2b, and M2c. M2a-polarized microglia are induced by IL-4 and IL-13, which activate the STAT6/IRF4 signaling pathway [56]. M2a-polarized microglia secrete the anti-inflammatory cytokine IL-10, and are characterized by the upregulation of arginase-1 (Arg-1), transglutaminase-2, RELM-*α*, and YM1, which antagonize M1-polarized microglia [61]. M2c-polarized microglia are induced by IL-10 and glucocorticoids, and are characterized by phagocytosis and its benefits, associated with clearing cell debris from the brain [62]. M2b-polarized microglia can also be induced by IL-1R ligands, and exhibit both anti-inflammatory and proinflammatory functions in the CNS, secreting IL-1*β*, TNF-*α*, and IL-10, an anti-inflammatory cytokine. M2b-polarized microglia have an M1 marker (CD86) and an M2 marker (IL-10^high^), and are therefore considered to be mixed-activation microglia [62–64]. It is important to remember that microglial phenotype descriptions are based on macrophage research, and microglia are not simply “macrophages in the CNS.” Microglial and macrophage polarization probably do not occur via completely the same mechanisms [65].

The phenotypes of reactive microglia occurring post-TBI have been well elucidated, but they are mainly based on focal cerebral injuries. Both M1- (indicated by CD86+/CD11b+) and M2- (indicated by CD206+/CD11b+) polarized microglia/macrophages increase significantly in CCI-induced injured brains post-TBI, peaking at 1 and 4 weeks post-TBI, respectively [66]. Most of the studies of microglial polarization after focal injury in different animal models have obtained similar results, with M2-polarized microglia increasing first and M1-polarized microglia persisting for longer periods of time [67–71]. The relationship between microglial polarization and TAI has been examined in fewer studies, but shows a similar trend to that during focal cerebral injury.

The number of M1-like microglia/macrophages in the corpus callosum was shown to increase during the first week after CCI-induced TBI in an animal model; their levels remained elevated for at least 14 days, as detected by reverse-transcription polymerase chain reaction (RT-PCR) and immunofluorescence staining. Abundant axonal injury in white matter, detected by immunohistochemical staining for neurofilament SMI-32, was strongly correlated with the number of M1-like phagocytes (markers: iNOS, CD11b, CD16, CD32, and CD86), whereas M2-like phagocytes (markers: CD206, Arg1, CCL-22, Ym1/2, and IL-10) peaked at 5 days and decreased rapidly and significantly thereafter [72]. These changes are consistent with those reported in focal cerebral cortex injury, but with a slower phenotypic shift from M2 to M1 polarization. At 7 days after TBI, M1- and M2-polarized microglia/macrophages were found to have increased according to RT-PCR and immunofluorescent staining (M1 marker: CD16; M2 marker: CD206), with Iba-1 as a marker for activated microglia; however, more M1-polarized microglia were found in the corpus callosum, accompanied by upregulation of proinflammatory factors NO, TNF-*α*, and IL-6 [73]. These findings suggest that the M1 phenotype promotes TAI-associated neuroinflammation. However, some studies have reported earlier M1 polarization and related TAI. Reactions with TNF-*α*, a proinflammatory factor mainly secreted by M1-polarized microglia, have been detected in the lysosomes of microglia in the corpus callosum as early as 30 min post-TBI in rats; these reactions were correlated with secondary axonal injury, detected 1 h post-TBI [74]. Based on these findings, it appears that M1-polarized microglia/macrophages are activated quite early too, and closely associated with post-TBI axonal injury.

## 4. Traumatic Axonal Injury Treatment Targeting Microglia

To date, human and animal studies have shown a close relationship between microglial activation and TAI, suggesting that microglia are a possible target for the treatment of this disorder. After TBI, M2-polarized microglia tend to exert their protective effects mainly by releasing anti-inflammatory cytokines, promoting tissue repair and regeneration, and facilitating phagocytosis, whereas M1-polarized microglia primarily exhibit proinflammatory effects that lead to neuronal injury. Based on current studies of post-TBI focal cerebral injury, post-TBI microglial activation and polarization are similar in both white and grey matter. M2-polarized microglia increase first, but then decrease quickly, whereas M1-polarized microglia increase slowly and persist for longer periods of time.

Because M1 is more likely to lead to TAI post-TBI and M2 plays a neuroprotective role and improves axonal injury, anti-neuroinflammatory treatment methods that promote M2 polarization while inhibiting M1 polarization have been shown to enhance neural function after TBI. Therefore, microglial polarization could also be developed as a target therapy for TAI, which is an important pathological effect of TBI.

### 4.1. Peroxisome Proliferator-Activated Receptors (PPARs)

PPARs are ligand-activated transcription factors belonging to the nuclear hormone receptor superfamily [75]. At least three different types of PPAR have been identified, PPAR-*α*, PPAR-*β*, and PPAR-*γ* [75, 76]. PPAR regulates neuroinflammation in the CNS; in stroke, spinal cord injury, and neurodegenerative disease animal models, PPAR agonists have been shown to be effective at protecting neural functions [75, 76].

Both fenofibrate, a PPAR-*α* agonist, and pioglitazone, a PPAR-*γ* agonist, have been shown to attenuate microglia-derived, nitric oxide-induced axonal injury *in vitro* [77]. In another study, diffuse brain injury was induced in rats by lateral fluid percussion injury (LFPI), and rosiglitazone was administered to induce PPAR-*γ* activation, which promoted microglial polarization to the M2 phenotype, as shown using CD206 and YM1 markers [78]; in the acute phase, the M2 and M1 phenotypes were significantly increased and suppressed as shown by CD16 and CD86 markers, respectively. TAI was shown to be relieved through the detection of *β*-APP and Bielschowsky's silver staining at 24, 48, and 72 h after injury, and neurological outcomes improved. Conversely, GW9662, a PPAR-*γ* antagonist, inhibited microglial polarization toward the M2 phenotype and aggravated inflammation, with more severe axonal injury and poorer neurological outcomes [78].

### 4.2. Minocycline

Minocycline is a classical antibiotic with anti-inflammatory and neuroprotective effects; it has been shown to inhibit M1 polarization and promote M2 polarization [79, 80]. Several animal studies have shown that minocycline improves neurological function by inhibiting post-TBI microglial activation [81–83]. However, the effects of minocycline on post-TBI TAI have not been elucidated.

The combined administration of minocycline and N-acetylcysteine in a mild-CCI rat model of TBI enhanced both M1- and M2-polarized microglia/macrophage activation in the corpus callosum at 2 days post-TBI, leading to the attenuation of white matter damage and improvement of neurological function [84]. However, some studies have reported contrasting results. In weight reduction-induced TBI in mice, minocycline was shown to reduce focal cortex injury by attenuating microglial activation by 59% at 24 h post-TBI, as detected by CD11b. Moreover, TBI-induced locomotor activity was relieved at 48 h post-TBI. However, axonal injuries evaluated by *β*-APP at 24 h post-TBI were unaffected [85]. These findings are consistent with those reported in a study of abuse head trauma (AHT) in a neonatal rat model, that acute minocycline administration decreased microglial/macrophage activation in the corpus callosum, such that effects were abolished by 7 days post-TBI; however, axonal injury, cerebral atrophy, and neurological function were not improved [86]. Extending the minocycline administration period to 9 days post-TBI led to even more severe neurodegeneration in the group with poorer neurological function [87].

A clinical trial of minocycline efficacy involving 15 patients with TBI was reported in 2018. Minocycline was shown to inhibit chronic microglial activation, as assessed using ^11^C-PBR28 PET; however, white matter damage and brain atrophy were markedly increased [43].

### 4.3. Erythropoietin

Several studies have reported that erythropoietin (EPO) administration protects neurological function post-TBI [88, 89]. One mechanism for this phenomenon is the regulation of inflammation through microglial/macrophage polarization toward the M2 phenotype [90–92]. In a study examining the effects of EPO on TAI, rats subjected to diffuse TAI followed by 30 min of hypoxia were administered with recombinant human EPO-*α* (5000 IU/kg); EPO was found to reduce axonal injury in white matter and enhance sensorimotor and cognitive recovery, as detected by NF-200, and low levels of activated microglia/macrophages (CD68+) and IL-1*β* were detected [93]. These results revealed that TAI could be attenuated by EPO therapy, partly through the modulation of microglia/macrophage activation.

### 4.4. SMM-189

SMM-189 is a cannabinoid type 2 (CB2) receptor inverse agonist. SMM-189 has been shown to convert human microglia from the M1 to the M2 phenotype *in vivo* [94]. In that study, a mild TBI mouse model was established using a focal left-side cranial blast; mild TBI was accompanied by widespread axonal injury surrounded by activated microglia (Iba-1+), mainly in the medial lemniscus, lateral lemniscus, cerebellar peduncles, deep cerebellar white matter, and pyramidal tract. SMM-189 has also been shown to partially reverse TBI-induced neural dysfunction including motor, visual, and emotional deficits by attenuating axonal injury. Moreover, a later study reported the effects of SMM-189 on the modulation of microglial polarization *in vivo* after mild TBI [95].

### 4.5. Progranulin (PGRN)

PGRN is a multifunctional growth factor involved in neuroinflammation with protective effects against neurodegenerative diseases [96]. Microglia/macrophage marker expression was compared between wild-type (WT) and granulin-deficient mice post-TBI; the results suggested that PGRN is produced in CD68-positive microglia and suppresses excessive inflammatory responses related to activated microglia after TBI, as detected by Iba-1, CD68, and CD11b [97, 98]. PGRN expression was found to be suppressed after spinal cord injury, whereas its immunoreactivity was colocalized with activated microglia/macrophages (CD68 and CD11b positive), indicating the close relationship between PGRN and microglia/macrophage activation [99]. PGRN-deficient mice were found to exhibit more obvious perilesional axonal injury than WT mice, despite similar overall brain tissue loss and neurological outcomes, with similar numbers of activated microglia/macrophages (Iba-1+) in both groups [100]. However, the expression levels of pro- and anti-inflammatory cytokines were elevated and suppressed, respectively, in PGRN-deficient mice. These findings suggest that PGRN modulates the polarization of microglia/macrophages toward the M2 phenotype while attenuating the associated TAI.

### 4.6. Laquinimod and Fingolimod

Laquinimod is an orally administered immunomodulator that was initially developed to treat multiple sclerosis (MS). In some animal models of diseases such as experimental autoimmune encephalomyelitis (EAE), laquinimod has been shown to inhibit microglial activation and attenuate axonal injury in CNS [101, 102]. In a moderate TBI study, laquinimod was administered to mice before and after TBI, and the brains were collected at 3 and 120 days post-TBI. Laquinimod was found to decrease axonal injury, enhance neurogenesis at an early stage, and prevent cerebral atrophy. Laquinimod also decreased monocyte infiltration into the brain, and further gene expression analysis revealed that it inhibited post-TBI microglial activation [103].

Fingolimod is an immunosuppressive synthetic compound produced by modifying metabolites extracted from *Isaria sinclairi* [104]; like laquinimod, it was initially developed to treat MS. Post-CCI fingolimod administration in mice was found to attenuate activated microglia/macrophages, as detected by Iba-1, and to augment the M2/M1 ratio and decrease axonal damage; treated mice also showed decreased immunoinflammatory response and improved neurological deficits post-TBI [105]. However, another study showed that administering fingolimod to suppress post-TBI inflammation did not improve neurological function [106].

### 4.7. Estrogen and Progesterone

Although the effects of sex on TBI outcomes have not been elucidated, studies have shown that the female sex hormones progesterone and estrogen suppress brain injury and improve neurological function [107–112]. Following diffuse brain injury in rats, progesterone administration reduced axonal and neuronal injury and markedly attenuated caspase-3 immunoreactivity [113]. Post-TBI administration of female sex steroids has been shown to induce anti-neuroinflammatory effects by modulating microglia/macrophage activation, producing neural protective effects [112, 114]. For example, in a CCI model of TBI, postovariectomy female mice with severe cerebral injuries at multiple brain sites showed enhanced microglial activation compared to intact females, as indicated by Iba-1 [107]. In post-TBI rats, the G1 agonist of estrogen receptor GPR30 was found to improve M2 polarization, as indicated by Arg1 and IL-4, leading to induced neural protection [115].

### 4.8. Omega-3 Polyunsaturated Fatty Acids (*ω*-3 PUFAs)

Several *ω*-3 PUFAs, including docosahexaenoic acid (DHA), *α*-linolenic acid, and eicosapentaenoic acid, play key roles in human metabolism [116]. Recent studies have shown that *ω*-3 PUFAs regulate post-TBI inflammatory and immune responses [117, 118]. In a CCI-induced TBI mouse model, DHA administration was found to inhibit microglia/macrophage activation, as detected by Iba-1, and decrease accumulation of *β*-APP; reduced neurofilament light levels in plasma at 28 days also indicated axonal injury attenuation [119]. Several studies have also reported that *ω*-3 PUFAs inhibit post-TBI neuroinflammation by suppressing microglia/macrophage activation and promoting polarization toward the M2 phenotype [120–124].

### 4.9. Histone Deacetylase Inhibition (HDAC)

The HDAC inhibitor 4-dimethylamino-N-(5-(2-mercaptoacetylamino)pentyl)benzamide (DMA-PB) has been reported as a potential therapy for inhibiting neuroinflammation by suppressing post-TBI microglial activation, as detected by OX-42 [125]. In a closed TBI mouse model, DMA-PB was confirmed to improve neurological function and decrease cerebral damage [126]. Administration of the HDAC inhibitor Scriptaid to a post-CCI mouse model showed that it enhanced long-term white matter preservation following TBI; damage to axons and the myelin sheath of the corpus callosum and striatum was assessed by a loss of myelin basic protein and an increase in abnormally dephosphorylated neurofilament protein, and by measuring the loss of myelin [73]. Scriptaid has also been shown to promote microglial polarization toward the M2 phenotype both *in vitro* and *in vivo*, as evaluated by CD206 detection and increased IL-10 gene expression, in a process modulated by the PI3K/Akt signaling pathway [73, 127].

### 4.10. Suppressors of Cytokine Signaling (SOCS)

SOCS proteins and cytokines comprise a family of intracellular proteins that are major negative regulators of the JAK/STAT pathway [128]. After mild TBI, SOCS2 overexpressing transgenic (SOCS2Tg) mice showed functional improvement, with lower cerebral lesion volume than control mice; after moderate TBI, levels of M2-polarized microglia/macrophages were increased around the cerebral injury lesion, as detected by CD206, whereas no difference in M1-polarized cell levels was detected by CD16/32 [129]. Other SOCS family members have been shown to inhibit inflammation by modulating microglial/macrophage polarization, and several TBI therapies such as hypothermia and melatonin have been found to be associated with these effects [130–132].

### 4.11. Complement Components

Several complement components including C1q, C3, and CR3 regulate microglia-synapse interactions following TBI; the accumulation of these components leads to chronic microglia/macrophage activation [133, 134]. The membrane attack complex (MAC) of the complement system is detectable in the traumatized brain soon after TBI. In post-TBI mice, blocking MAC by inhibiting C6 was shown to reduce axonal loss and promote neurologic function, with decreased microglia/macrophage accumulation, detected by Iba-1 [135]. Blocking MAC has also been shown to inhibit post-TBI chronic inflammation by suppressing microglia/macrophage activation, as detected by Iba-1 [134].

### 4.12. IL-1 Receptor Antagonist (IL-1ra)

IL-1 is a classic proinflammatory cytokine whose expression is significantly elevated post-TBI [136]. M1 phenotype microglia/macrophages are a major source of IL-1 post-TBI. Post-TBI administration of IL-1ra has been shown to induce anti-inflammatory properties and promote neurological function [137–139] by inhibiting microglia/macrophage activation. IL-1ra administration has also been shown to decrease microglial activation, as detected by Iba-1, and cerebral edema during the acute phase of TBI in mice, and to attenuate chronic-phase TAI [140]. A single-center randomized clinical trial (RCT) of IL-1ra reported its safety, good brain penetration, and ability to modify neuroinflammatory reactions, demonstrating its potential as a neural protective agent [141]. IL-1ra administration has also been shown to increase cytokines, as detected by granulocyte-monocyte colony stimulating factor (GM-CSF) and IL-1*β*, and M1-polarized microglia, indicating that it may be insufficient to classify IL-1ra as merely an anti-inflammatory cytokine [142].

### 4.13. Stem Cells

Stem cell transplantation is a potential therapy for several neural system diseases, including TBI. Stem cells have been shown to regulate the activation and polarization of microglia/macrophages, mainly by inducing a shift toward the M2 phenotype [143–147], which is likely to be one of the main mechanisms for this therapy. Stem cell therapy has been demonstrated to improve TBI outcomes effectively in some clinical trials [148–151].

In a severe CCI-induced TBI model, the transplantation of neural stem cells (NSC) was reported to reduce the intensity of activated microglia significantly, as detected by Iba-1, and promote microglial polarization from the M1 to the M2 phenotype, especially around the axonal injury sites. These changes were accompanied by reduced axonal injury, but without decreasing the volume of cerebral contusions [143]. Similar results were reported in a study using human amnion epithelial cells and bone marrow mesenchymal stromal cells to treat TBI in rats [152–154].

Stem cell therapy is inhibited by reduced BBB penetration efficiency. Exosomes, which are extracellular vesicles secreted from cells, containing noncoding RNA, proteins, and other small substances, can easily pass through the BBB. Several studies have shown that exosomes secreted by stem cells can modulate microglial/macrophage polarization [68, 155], and may be more effective when injected through peripheral vessels.

### 4.14. Hypothermia

Hypothermia, which can reduce intracranial pressure and has neural protective effects, is a popular therapeutic option for severe TBI. Hypothermia has been shown to attenuate TAI in different TBI animal models [156–159]. The protective mechanisms of hypothermia include decreasing cerebral oxygen metabolism, inhibiting internal harmful cytokines, preventing apoptosis and, most importantly, inhibiting neuroinflammation [160, 161]. In a disease model of hypoxic-ischemic encephalopathy, hypothermia was shown to attenuate axonal injury by inhibiting microglial activation, as detected by Iba-1 [162]. Another TBI study showed that the ratio of M1- to M2-polarized microglia was significantly lower in the cortical and hippocampus regions after 24 h of hypothermia therapy at 33°C than in the control group [70].

Besides, several other therapies, including clofazimine, a Kv1.3 channel blockade [163], lacosamide [164], and vascular endothelial growth factor- (VEGF-) C [165], have been shown to improve TAI by targeting the activation and polarization of microglia/macrophages.

## 5. Limitations and Future Directions

Though recent studies have shown the capacity of microglia as potential drug targets for TAI, there are still several limitations. The effects of microglial activation are complex; for example, conflicting reports on the effects of minocycline on patient outcomes [43, 84, 85] indicate that simply inhibiting microglia/macrophage activation may not be effective in improving patient outcomes. Similar results were revealed in several other studies. For example, CD11b-TK (thymidine kinase) mice developed as a valganciclovir-inducible model of microglia/macrophage deletion were used to establish a closed-skull TBI model; at 7 days post injury, valganciclovir was found to reduce the microglia/macrophage population dramatically in the corpus callosum and external capsule, but it did not attenuate axonal injury, as detected by silver staining, APP accumulation, neurofilament labeling, and electron microscopy. Longer treatment with valganciclovir even led to neural toxic effects [166].

Moreover, microglial activation is a long-term phenomenon following TBI. The close relationship between axonal injury and microglial activation can be detected several years post-TBI. As a result, transient therapy targeting microglia may not effectively protect against axonal injury. Acute minocycline administration, which affects microglia only transiently, did not attenuate cerebral injuries including TAI [86].

Besides, most studies observe microglial responses using macrophage markers or gene expression. Although axonal injury occurs mainly in the corpus callosum, internal capsule, and brain stem, where few macrophages can infiltrate through the intact BBB, it remains difficult to differentiate infiltrating macrophages and activated microglia after TBI.

Based on the findings of recent studies, the ideal method for targeting microglia to attenuate TAI is to promote microglial polarization to the M2 phenotype (anti-inflammation) and to inhibit polarization to the M1 phenotype (proinflammation) at the appropriate times. However, it is difficult to describe all types of polarized microglia as simply pro- or anti-inflammation. The terminology used to describe microglial phenotypes is based on macrophage research, and is probably insufficient to discuss macrophages in the CNS; a new classification system for microglia should be developed in a future study. Nevertheless, despite these controversies and limitations, the body of literature supports targeting microglia as a potential TAI therapy.

## 6. Conclusion

TAI is a major cause of death and severe disability secondary to TBI; however, effective treatments remain limited. Recent studies suggest that post-TBI microglial activation plays an important role in the pathology of axonal injury, and that neuroinflammation, which is closely related to post-TBI microglial activation, is a potential target for pharmaceutical therapy. However, simply inhibiting microglial activation may be insufficient for attenuating axonal injury.

In addition to its neuroinflammatory effects, activated microglia have shown neural protective effects. Potential TAI treatments could target microglia by regulating the phenotype of activated microglia at appropriate times. The complex nature of microglial phenotype alteration requires further investigation to determine the optimal intervention time. Active microglia persist in white matter for long periods after TBI, and are probably related to various neural degenerative diseases. Treatment endpoints also require further investigation.

## Data Availability

The data supporting this review are from previously reported studies which have been cited.
